# Editorial: Combating Redox Imbalance-Associated Complications With Natural Products

**DOI:** 10.3389/fphar.2021.802750

**Published:** 2021-11-26

**Authors:** Abhay K. Pandey, Shashank Kumar, Akhilesh K. Pandey, Flávio Reis

**Affiliations:** ^1^ Department of Biochemistry, University of Allahabad, Prayagraj, India; ^2^ Department of Biochemistry, School of Basic Science, Central University of Punjab, Bathinda, India; ^3^ Texas Tech University Health Sciences Center, Lubbock, TX, United States; ^4^ Coimbra Institute for Clinical and Biomedical Research (iCBR), Faculty of Medicine, University of Coimbra, Coimbra, Portugal; ^5^ Center for Innovative Biomedicine and Biotechnology (CIBB), University of Coimbra, Coimbra, Portugal; ^6^ Clinical Academic Center of Coimbra (CACC), Coimbra, Portugal

**Keywords:** redox homeostasis, oxidative stress, natural products, chronic diseases, free radicals

Living cells continuously produce reactive oxygen species (ROS) as byproduct during cellular metabolic processes and also utilize them as signaling molecules (Schieber and Chandel, 2014). Sometimes, the production of ROS increases at an alarming rate due to varying reasons. The body has evolved different response mechanisms to combat increased ROS. Ultimately, in a feedback mechanism, ROS-mediated oxidative stress is mitigated by a variety of biological processes at molecular level such as apoptosis, autophagy, and necrosis. Increased and uncontrolled ROS generation is known to damage cellular structures/organelles and produces inflammatory response that predisposes to various diseases and ailments including cardiovascular, aging, neuro-degeneration, diabetes, and cancer, among others (Milkovic et al., 2019). Living organisms have evolved a complex redox system comprising enzymatic and non-enzymatic antioxidant systems to maintain a physiological equilibrium between oxidative stress and antioxidant defense (He et al., 2017). This equilibrium maintains the redox homeostasis and allows the physiological process to occur at normal pace. ROS production at lower levels is beneficial to the body as it succors immunity, signaling pathways, pathogen killing, wound healing, etc. Natural products are known to possess disease preventive as well as therapeutic attributes that substantiate their importance in human life (Kumar and Pandey, 2013). Habitat flora has always provided necessary compounds and drugs with preventive/therapeutic efficacy for the survival advantage against oxidative stress and lifestyle diseases. Moreover, the use of natural products is also associated with less side effects and cost effectiveness coupled with enormous diversity of chemical entities. The present research topic “Combating Redox Imbalance-Associated Complications with Natural Products” has gathered five articles which include critical reviews and original research articles contributed by about 44 potential researchers working in the field of redox imbalance-associated complications and their mitigation by natural products.

Accumulation of lipids (especially of cholesterol) in macrophages in the large/medium size arteries is the main pathophysiological event in atherosclerosis, with oxidized low-density lipoproteins (oxLDL) presenting a central role in early onset and disease progression (Obermayer et al., 2018). In this context, Poznyak et al. discussed the role of oxLDL in atherosclerosis at various stages of the disease. The authors reviewed the oxidative stress-mediated emergence of oxLDL, which is crucial for the progression of cardiovascular disease (CVD)-linked atherosclerosis. The review beautifully presented the biochemistry of LDL oxidation and its involvement in intracellular cholesterol estimation which leads to associated pathophysiology. OxLDL binds to LOX-1 membrane receptor and triggers various cellular events such as oxLDL uptake, apoptosis, foam cell formation, and endothelial activation. Autoantibody generation, scavenger receptors, and 12/15-lipooxygenase activity are shown to be associated with the LDL oxidation-mediated atherosclerosis lesions formation/amelioration. Thus, this review highlights the importance of oxLDL as therapeutic target for controlling atherosclerosis using anti-oxLDL antibodies. The authors also discussed the putative role of miRNAs and antioxidant enzymes in oxLDL formation and associated pathology.

Genetic constitution and life-time exposures determine the phenotype of an individual in a combined way. Evolution of animals in an oxygen-rich environment created a redox interface between an individual and its environment. The redox players are present in the body at almost all the metabolic/structural system levels, playing a major role in the response to environmental exposures and other physiological challenges during the life span. Accumulation of oxidative stress inducers throughout the life span ultimately disrupts the redox network in the body and leads to aging. Exploring the role of antioxidants as therapeutic intervention in delaying ovarian aging, Yang et al. presented various theories proposed for ovarian aging and also discussed the cellular and molecular mechanisms putatively involved, including free radicals, stress-mediated apoptosis in ovaries, shortening of telomere, mitochondrial dysfunction, and inflammation. Furthermore, the review highlights the oxidative stress modulatory role of potential phytochemicals (hyperoside, crocetin, genistein, proanthocyanidin, quercetin, resveratrol, curcumin, crocin, catalpol, mogroside V, and icariin) and their promising role in physiological/molecular events in the mitigation of ovary aging. Natural products possess anti-ovarian aging potential by virtue of their improved mitochondrial function and anti-inflammatory, antioxidant, anti-apoptosis, and telomere protection ability (Wang et al., 2019). The review provides an insight in overcoming age-related fertility issues in reproductive medicine.

Systemic oxidative stress is one of the important causes of menopause-related complications in women including chronic kidney disease (CKD). Patients on hormonal replacement therapy show various side effects of the therapy that includes breast cancer and other critical pathological conditions in menopausal women (Chlebowski et al., 2020). The underlying mechanism and proper treatment of menopause-induced CKD is not yet clear, requiring further research on the topic. The original article by Liu et al. reported the medicinal efficacy of *Hypericum perforatum* L. extract in ovariectomy (OVX)-induced kidney dysfunction in an experimental model. The study reports the *in vivo* experimentation and omics approach to find the underlying anti-CKD mechanism of *H. perforatum* extracts. The study tried to link glutathione redox imbalance with the kidney impairment. The extract showed anti-CKD activity by maintaining the cellular redox homeostasis. The test extract also altered the gamma-glutamyl transferase 1 (Ggt1) activity, efflux of toxic metabolites, alanyl-aminopeptidase, and other biochemical events which ultimately revealed that *H. perforatum* extracts have potential to prevent CKD by maintaining the redox homeostasis.

Photoaging is a detrimental phenomenon in which the skin structure damage occurs due to the exposure to UV radiation (UR) in the course of time. UR activates the enzymatic machinery and transcription factors which ultimately damage the collagen and leads to photoaging (Nakyai et al., 2017). Several biochemical, molecular, and physiological factors are associated with photoaging. Antioxidant system-mediated inhibition of UV-induced metalloproteinase-1 (MMP-1) activity is an important mechanism to target photoaging. Several studies reported antioxidant system (e.g., Nrf2 signaling)-mediated anti-photoaging potential in natural products. Lohakul et al. have reported the anti-photoaging property and the underlying mechanism of a Thai polyherbal formulation known as Harak. The product has been traditionally used by Thai countrymen for skin nourishment and mitigation of skin-related problems. The study discussed the role of nuclear factor E2-related factor 2 (Nrf2) in the regulation of phase II detoxifying enzymes such as glutathione S-transferase (GST) and NAD(P)H:quinone oxidoreductase-1 (NQO-1) involved in UR-mediated skin damage. It has been proposed that UR-induced oxidative stress or ROS production downregulates Nrf2 and thereby reduces the activity of phase II detoxifying enzymes which ultimately leads to increased MMP-1 activity and collagen damage. With the help of molecular and biochemical techniques (flow cytometry, western blot, qRT-PCR, microscopy, and immunofluorescence), authors studied the anti-photoaging mechanism of Harak using *in vitro* and *in vivo* experimental models. Results showed that Harak phytochemical(s) are able to ameliorate UV-mediated skin damage by upregulating the Nrf2 molecular events and decreasing the MMP-1 activity.

Neurodegenerative diseases involve complications majorly characterized by immune dysfunction where brain immune cell (i.e., microglia) plays an important role (Biber et al., 2019). Pharmacological targeting of microglia has been shown to reduce redox imbalance and neuroinflammation, which in turn potentially regulate the progression of neurological diseases like Alzheimer's, Parkinson's, and multiple sclerosis (Liu et al., 2019). The review article by Maurya et al. presented extensive data mining that suggested the use of natural products (curcumin, resveratrol, cannabidiol, ginsenosides, flavonoids, and sulphoraphane) in reducing neuronal toxicity, inflammation, oxidative stress, and microglia activation during neurodegeneration process. In the present review, authors summarized the role of microglia and potential microglia-specific genes and proteins as targets for therapeutic purpose using natural products. Furthermore, the authors also presented the molecular docking of microglia-specific Iba1 protein with natural products which indicated that Iba1 could be a target of curcumin, cannabidiol, and resveratrol. The study suggested the precise drug screening and identification of molecular targets of microglia to combat neuroinflammation and redox imbalance in neurodegenerative disorders.
